# An Efficient Strategy Combining Immunoassays and Molecular Identification for the Investigation of *Fusarium* Infections in Ear Rot of Maize in Guizhou Province, China

**DOI:** 10.3389/fmicb.2022.849698

**Published:** 2022-03-14

**Authors:** Guofu Shang, Shuqin Li, Huan Yu, Jie Yang, Shimei Li, Yanqin Yu, Jianman Wang, Yun Wang, Zhu Zeng, Jingbo Zhang, Zuquan Hu

**Affiliations:** ^1^Key Laboratory of Infectious Immune and Antibody Engineering of Guizhou Province, School of Basic Medical Sciences/School of Biology and Engineering, Guizhou Medical University, Guiyang, China; ^2^Key Laboratory of Environmental Pollution Monitoring and Disease Control, Ministry of Education, Guizhou Medical University, Guiyang, China; ^3^Immune Cells and Antibody Engineering Research Center of Guizhou Province, Cellular Immunotherapy Engineering Research Center of Guizhou Province, Guizhou Medical University, Guiyang, China; ^4^State Key Laboratory of Functions and Applications of Medicinal Plants, Guizhou Medical University, Guiyang, China; ^5^Wheat Anti-toxin Breeding Laboratory, College of Plant Science and Technology, Huazhong Agricultural University, Wuhan, China

**Keywords:** *Fusarium* infections, enzyme-linked immunosorbent assay (ELISA), molecular identification, population structure, mycotoxin chemotype

## Abstract

*Fusarium* is one of the most important phytopathogenic and mycotoxigenic fungi that caused huge losses worldwide due to the decline of crop yield and quality. To systematically investigate the infections of *Fusarium* species in ear rot of maize in the Guizhou Province of China and analyze its population structure, 175 samples of rotted maize ears from 76 counties were tested by combining immunoassays and molecular identification. Immunoassay based on single-chain variable fragment (scFv) and alkaline phosphatase (AP) fusion protein was first employed to analyze these samples. *Fusarium* pathogens were isolated and purified from *Fusarium*-infected samples. Molecular identification was performed using the partial internal transcribed spacer (*ITS*) and translation elongation factor 1α (*TEF-1*α) sequences. Specific primers were used to detect toxigenic chemotypes, and verification was performed by liquid chromatography tandem mass spectrometry (LC–MS/MS). One-hundred and sixty three samples were characterized to be positive, and the infection rate was 93.14%. Sixteen species of *Fusarium* belonging to six species complexes were detected and *Fusarium meridionale* belonging to the *Fusarium graminearum* species complex (FGSC) was the dominant species. Polymerase chain reaction (PCR) identification illustrated that 69 isolates (56.10%) were potential mycotoxin-producing *Fusarium* pathogens. The key synthetic genes of NIV, NIV + ZEN, DON + ZEN, and FBs were detected in 3, 35, 7, and 24 isolates, respectively. A total of 86.11% of *F. meridionale* isolates carried both NIV- and ZEN-specific segments, while *Fusarium verticillioides* isolates mainly represented FBs chemotype. All the isolates carrying DON-producing fragments were FGSC. These results showed that there are different degrees of *Fusarium* infections in Guizhou Province and their species and toxigenic genotypes display regional distribution patterns. Therefore, scFv-AP fusion-based immunoassays could be conducted to efficiently investigate *Fusarium* infections and more attention and measures should be taken for mycotoxin contamination in this region.

## Introduction

Maize (*Zea mays* L.) is an important food and feed crop worldwide and one of the main crop in Guizhou Province, China. Numerous pathogenic fungi can infect maize and cause *Gibberella* ear and stalk rot, resulting in yield and quality reductions and a threat to global food security (Pechanova and Pechan, 2015). *Fusarium* is one of the most economically destructive and species-rich groups of large-scale pathogenic fungi in the world. These pathogens are present in various natural environments and can infect host crops throughout the growth cycle, causing seed rot, seedling rot, stem rot and panicle rot (Karlsson et al., 2021; Palacios et al., 2021). Fungal crop diseases not only seriously affect the growth and development of plants and product quality but also produce a variety of mycotoxins, such as nivalenol (NIV), zealenone (ZEN), deoxynivalenol (DON), and fumonisins (FBs) (Torres et al., 2019). More seriously, these mycotoxins are detrimental to human and animal health due to their serious acute toxicity, cytotoxicity, immunotoxicity, teratogenicity, mutagenicity and carcinogenicity (Lee and Ryu, 2017; Sun et al., 2017).

The production of food and feed crops in China mainly includes rice, wheat, and maize. *Fusarium* can invade at multiple stages of plant growth and is widely present in these important food crops (Torres et al., 2019; Palacios et al., 2021). *Fusarium* pathogens often appear in high-humidity and high-heat areas and *Fusarium* head blight (FHB) has reached historically high epidemic acreages in the middle and lower reaches of the Yangtze River (Wang et al., 2011; Yang et al., 2021). It is also one of the major diseases in wheat and maize planting areas in the Northeast Plain, North China Plain, and Sichuan Basin (Feng et al., 2011; Wei et al., 2013; Wang et al., 2021). Thus, *Fusarium* poses a great threat to China’s food production (Gong et al., 2009; Qiu and Shi, 2014; Qiu et al., 2019). Simultaneously, the suitable living environment of different *Fusarium* species varies and is influenced by ecological factors such as geography and climate. The dominant species of pathogens isolated in different countries, different regions, and different years display significant discrepancies (Zhang et al., 2007). Guizhou is located in the Yunnan-Kweichow Plateau, and the temperature is relatively low. Theoretically, it is not a high-incidence area of *Fusarium* diseases, and rarely are concerns focused on these regions. In recent years, some investigations have shown that *Fusarium* can cause plant diseases in this area, such as maize ear rot, *Pinellia* tuber rot, and tobacco root rot (Shi et al., 2015; Yu and Yao, 2018; Shang et al., 2019). However, the infections, distribution and mycotoxin production of *Fusarium* pathogens in this area have not been systematically investigated.

*Fusarium* species have some discrepancies in their housekeeping genes, and molecular identification based on these genes has been widely used for further identification of morphological *Fusarium* species. DNA sequences, such as translation elongation factor 1-α (*TEF-1*α), β-tubulin (β*-TUB*), calmodulin (*CAM*), mitochondrial small subunit rDNA (*mtSSU*), 28S rDNA, and internal transcribed spacer (*ITS*) regions, are widely used for phylogenetic analysis to assess the genetic relationships of *Fusarium* species (Wang et al., 2014b). O’Donnell et al. (2013, 2015) divided *Fusarium* into 20 species complexes based on Genealogical Concordance Phylogenetic Species Recognition (GCPSR) analyses of more than 300 pathogens, which provided the basis for the identification and classification of *Fusarium* species based on DNA sequences. Unfortunately, the pretreatment of molecular identification is labor intensive and time-consuming because the pathogenic fungi in each sample must be subcultured, purified, and polymerase chain reaction (PCR) amplified (Santos et al., 2016; Cambaza et al., 2019; Schiwek et al., 2020). Enzyme-linked immunosorbent assay (ELISA) has distinctive characteristics of simple operation, low price, high sensitivity, good specificity and simple pretreatment of samples (Saccon et al., 2017; Rahman et al., 2019). At present, many fungi-specific antibodies have been exploited to develop immunoassays for rapid detection and monitoring of fungal infections (Wang et al., 2017; He et al., 2018). Therefore, the application of immunoassays for preliminary screening of *Fusarium*-infected samples is more efficient and convenient for further molecular identification. In our previous study, a single chain variable fragment (scFv) named FvSG7 has been selected from a phage display library and its fusion protein with alkaline phosphatase (FvSG7-AP) has been verified to efficiently detect *Fusarium* pathogens in cereal grains (Hu et al., 2013). Therefore, this study aimed to first investigate the *Fusarium* infections in maize and geographic distribution in Guizhou Province of China by using the established rapid immunoassay method. Furthermore, their population structure and toxigenic chemotypes were analyzed by PCR identification and liquid chromatography tandem mass spectrometry (LC–MS/MS) detection. Our results will lay a foundation for effective identification of *Fusarium* pathogens in the field and further understanding the distribution characteristics of *Gibberella* ear rot and mycotoxin chemotypes.

## Materials and Methods

### Experimental Materials

Diseased maize ears with similar symptoms, including kernels covered with white, pink or salmon-colored mold or exhibiting a white streaking (“starburst”) symptom, were collected from counties in Guizhou Province, China, in the maize-harvesting period.

In order to quickly analyze the *Fusarium* infections in ear rot of maize samples, FvSG7-AP fusion was used for *Fusarium* detection with one-step ELISA. The recombinant *Escherichia coli* strain XL1-Blue/pDAP2/S-FvSG7 was obtained by transforming the recombinant plasmid pDAP2/S-FvSG7 containing the *FvSG7-AP* fusion gene into *E. coli* XL1-Blue competent cells. The *FvSG7* gene (GenBank accession number KC304795) encodes an anti-*Fusarium* scFv antibody isolated previously (Hu et al., 2013), and the pDAP2/S vector contains a gene encoding AP enzyme (Kerschbaumer et al., 1997).

### Expression of FvSG7-AP Fusion Protein

Twenty microliters of recombinant strain XL1-Blue/pDAP2/S-FvSG7 were inoculated into 20 mL of 2 × TY medium (16 g/L tryptone, 10 g/L yeast extract, and 5 g/L NaCl, pH 7.0) supplemented with 100 μg/mL Amp. After incubation overnight at 37°C and 200 r/min, 10 mL of the culture was inoculated into 200 mL of 2 × TY medium supplemented with 100 μg/mL Amp and cultured at 37°C and 200 r/min until the OD_600 nm_ reached 0.5–0.6. A final concentration of 0.1 mmol/L isopropyl-β-D-thiogalactopyranoside (IPTG) was added for 20 h of induction at 16°C and 200 r/min. The FvSG7-AP fusion protein was extracted by ultrasonication, and the enzyme activity of AP was tested using *p*-nitrophenyl phosphate (pNPP) solution (Wang et al., 2015). The fusion protein was purified by Ni-NTA chromatography, and its concentration was determined by the Brandford method.

### Immunoassay Detection of Samples

The collected maize samples were crushed to power and detected by using ELISA method based on the FvSG7-AP fusion protein (Hu et al., 2013). In detail, 0.2 g of each sample was weighed and transferred to 1.5-mL Eppendorf tubes. Then, 1 mL of phosphate-buffered saline (PBS) was added and incubated for 30 min at room temperature with shaking. The homogenates were left standing for 10 min, and then, 100 μL of supernatant was pipetted into the ELISA plate wells. After incubation at 37°C for 2 h, the wells were washed three times with PBS buffer. Next, 200 μL of 2% skimmed milk was added to each well and incubated at 37°C for 2 h. After three washes with PBS, 100 μL of purified FvSG7-AP fusion protein was added to each well. The plates were placed in a 37°C incubator for 1.5 h and washed three times with PBST (PBS containing 0.1% Tween-20) and PBS buffer. Finally, 100 μL of 0.2% pNPP solution was added, and the absorbance was recorded at 405 nm by a microplate reader. Negative controls coated with healthy maize were set up, and each example was repeated in three wells.

### Isolation of *Fusarium* Pathogens From Maize Kernels

Symptomatic kernels were soaked in 70% alcohol for 30 s and transferred into 2% sodium hypochlorite solution for another 2 min of immersion. After five washes with sterile water, the kernels were dried on sterile filter paper. Then, each seed was cut in half and placed on PDA medium for a 5-day incubation at 28°C in the dark. The mycelia were observed under a microscope, and colonies displaying morphological characteristics of *Fusarium* were subcultured onto fresh PDA medium (Nelson et al., 1983; Leslie and Summerell, 2006). The putative *Fusarium* colonies were purified using a single-sporing method.

### Genomic DNA Extraction and PCR Amplification

Each isolate was inoculated on fresh PDA medium and cultured at a constant temperature of 28°C for 5 days, and then, the mycelial mass was harvested by scraping. The genomic DNA of *Fusarium* pathogens was extracted by using a fungal genomic DNA extraction kit (Solarbio, Beijing, China) and stored at −20°C. PCR amplification and sequencing of the *ITS* and *TEF-1*α genes were achieved using the primer pairs ITS4/ITS5 (White et al., 1990) and EF1T/EF2T (Mirete et al., 2004), respectively (Table 1). The PCR products were detected by 1% agarose gel electrophoresis and sequenced by Sangon Biological (Shanghai) Co., Ltd.

**TABLE 1:** Information about primers used in the experiments.

Primer name	Primer sequence (5′-3′)	Expected size (bp)	Gene	References
ITS5	GGAAGTAAAAGTCGTAACAAGG	550	*ITS*	White et al., 1990
ITS4	TCCTCCGCTTATTGATATGC			
EF1T	ATGGGTAAGGAGGACAAGAC	648	*TEF-1*α	Mirete et al., 2004
EF2T	GGAAGTACCAGTGATCATGTT			
FUM5F	GTCGAGTTGTTGACCACTGCG	845	*FUM1* (FBs)	Bluhm et al., 2002
FUM5R	CGTATCGTCAGCATGATGTAGC			
FUM1F	ATTATGGGCATCTTACCTGGAT	798	*FUM1* (FBs)	Ramana et al., 2011
FUM1R	ACGCAAGCTCCTGTGACAGA			
FUM13F	AGTCGGGGTCAAGAGCTTGT	988	*FUM13* (FBs)	
FUM13R	TGCTGAGCCGACATCATAATC			
PKS4F	AGCAGCAATAAGAACCAG	1,076	*PKS4* (ZEN)	Zhang et al., 2009
PKS4R	GACACTTCCAACCCACAG			
PKS4F	CGTCTTCGAGAAGATGACAT	280	*PKS4* (ZEN)	Sim et al., 2018
PKS4R	TGTTCTGCAAGCACTCCGA			
PKS13F	CTGAGAAATATCGCTACACTACCGAC	192	*PKS13* (ZEN)	
PKS13R	CCCACTCAGGTTGATTTCGTC			
ZEB1F	AAATAATTTACCCGTTCTTCTGGGAACT	129	*ZEB1* (ZEN)	
ZEB1R	CTGAAACGGAGGTGTTGAGG			
ZEB2F	GGGATTAACCGCTGTGG	80	*ZEB2* (ZEN)	
ZEB2R	TAGGCATGCCCGAAACCGAAAGT			
TRI13F	TACGTGAAACATTGTTGGC	234 (DON) 415 (NIV)	*Tri13*	Li et al., 2009
TRI13R	GGTGTCCCAGGATCTGCG			
TOXP1	GCCGTGGGGRTAAAAGTCAAA	300 (DON) 360 (NIV)	*Tri5-Tri6*	Li et al., 2005
TOXP2	TGACAAGTCCGGTCGCACTAGCA			

### Phylogenetic Analysis

DNA sequences were aligned and adjusted manually using DNAStar-SeqMan software^1^. Sequence similarity searches were performed with the BLAST network service based on the FUSARIUM-ID database^2^ and NCBI GenBank.^3^ NRRL, MRC or CBS strains were taken as standard controls, and their *TEF-1*α sequences were downloaded (Supplementary Table 1). Phylogenetic analysis was performed with MEGA-X-10.0.5 software (Kumar et al., 2018), and phylogenetic trees were constructed by using the maximum-likelihood method. Numbers above branches were signed to indicate bootstrap values based on 1,000 replications. The best-fit model of molecular evolution was selected based on the estimation of Bayesian information criterion scores.

### Molecular Identification of Toxigenic Chemotypes

Single and/or multiplex PCR were performed to analyze the genes involved in NIV, ZEN, DON, and FBs synthesis using chemotype-specific primer pairs (Table 1). Briefly, PCR amplification was conducted in a final volume of 25 μL containing 1 μL of genomic DNA, 1 μL of each primer (10 μmol/L), 12.5 μL of 2 × *Taq* PCR starMix kit (GenStar, Beijing, China). A negative control omitting DNA template was used in every set of reactions. The PCR products were detected by 1% agarose gel electrophoresis. Each toxigenic chemotype was identified using two or more primer pairs and repeated three times.

### Liquid Chromatography Tandem Mass Spectrometry Detection of Mycotoxin Production

Two *Fusarium* isolates of each toxigenic chemotype (NIV: LPS-LZ-01, ZY-SY-01; ZEN: QXN-XR-01, GY-GSH-01; DON: QXN-ZF-01, BJ-HZ-02; and FBs: GY-XF-02, QN-LD-03) were selected as representative isolates. Also, *Fusarium miscanthi* represent isolates of GY-HX-03 and GY-HX-212 and *Fusarium concentricum* isolate QDN-RJ-01 were cultured. The mycotoxin production was measured by LC–MS/MS and repeated twice. In detail, the *Fusarium* isolates were inoculated on PDA and cultured at 28°C in the dark for 3–5 days. For NIV and ZEN, 4 mycelium-agar plugs with a 5-mm diameter were placed in a sterilized conical flask containing niblet medium culture (100 g of niblet, 50 mL of deionized water) and statically cultured at 28°C in the dark for 2 weeks. For DON, the mycelia were inoculated in half-strength CM-cellulose-yeast extract (CMC) broth to prepare the spore suspension (Xu et al., 2010), and then, 1 mL of spore fluid was inoculated into the niblet medium culture and statically cultured at 28°C in the dark for 2 weeks. For FBs, the mycelia were inoculated into a sterilized conical flask containing mung bean broth (2 g of mung bean, 200 mL of deionized water) to prepare the spore suspension, and then, 1 mL of spore fluid was inoculated into niblet medium culture for 3 weeks. Grains were dried in a 55°C incubator and finely ground to powder. Five grams of the samples were weighed and supplemented with 10 mL of water and 10 mL of acetonitrile containing 10% formic acid. After treatment with ultrasonication for 15 min, 1 g of citric acid, 1 g of NaCl and 4 g of MgSO_4_ were added. The mixture was vortexed for a few seconds, followed by centrifugation at 5,000 r/min for 5 min. Then, 5 mL of supernatant was pipetted into a tube, and 0.75 g of MgSO_4_, 0.25 g of primary secondary amine (PSA) and 0.15 g of octadecyl silane bonded phase (C18) were added, mixed and centrifuged at 5,000 r/min for 5 min. For each sample, 0.5 mL of the supernatant was pipetted, and 0.5 mL of 1% formic acid water was added. Subsequently, the fluid was passed through 0.22-μm nylon filters and quantified using an external standard method at Zhongke Youlong (Hangzhou) Food Safety Standard Technology Co., Ltd.

Chromatographic separation was studied on CNW Athena ultra high performance liquid chromatography (UHPLC) C18 Column (100 mm × 2.1 mm, 1.8 μm). The flow rate was set as 0.25 mL/min; the injection volume was 10.0 μL; and the column temperature was kept constant at 35°C. The mobile phase consisted of water containing 0.1% formic acid (A) and acetonitrile (B). The gradient elution program was performed as follows: 5% B from 0 to 2 min, 5–95% B from 2 to 12 min, 95–99% B from 12 to 12.1 min, 99% B from 12.1 to 14 min, 99–5% B from 14 to 14.1 min, 5% B from 14 to 16 min. The injection volume was 10 μL. MS/MS detection was performed on a triple quadrupled mass spectrometer detector equipped with a jet stream electrospray ionization (ESI) source under multi-reaction monitoring (MRM) conditions. ESI positive (ESI^+^) and negative (ESI^–^) subsection acquisition modes were used for the quantification with a capillary voltage of 5.5 kV. The specific MS parameters for mycotoxin analyses in this study are displayed in Supplementary Table 2.

## Results

### Expression of Fusion Protein and Enzyme-Linked Immunosorbent Assay Detection

**FIGURE 1:**
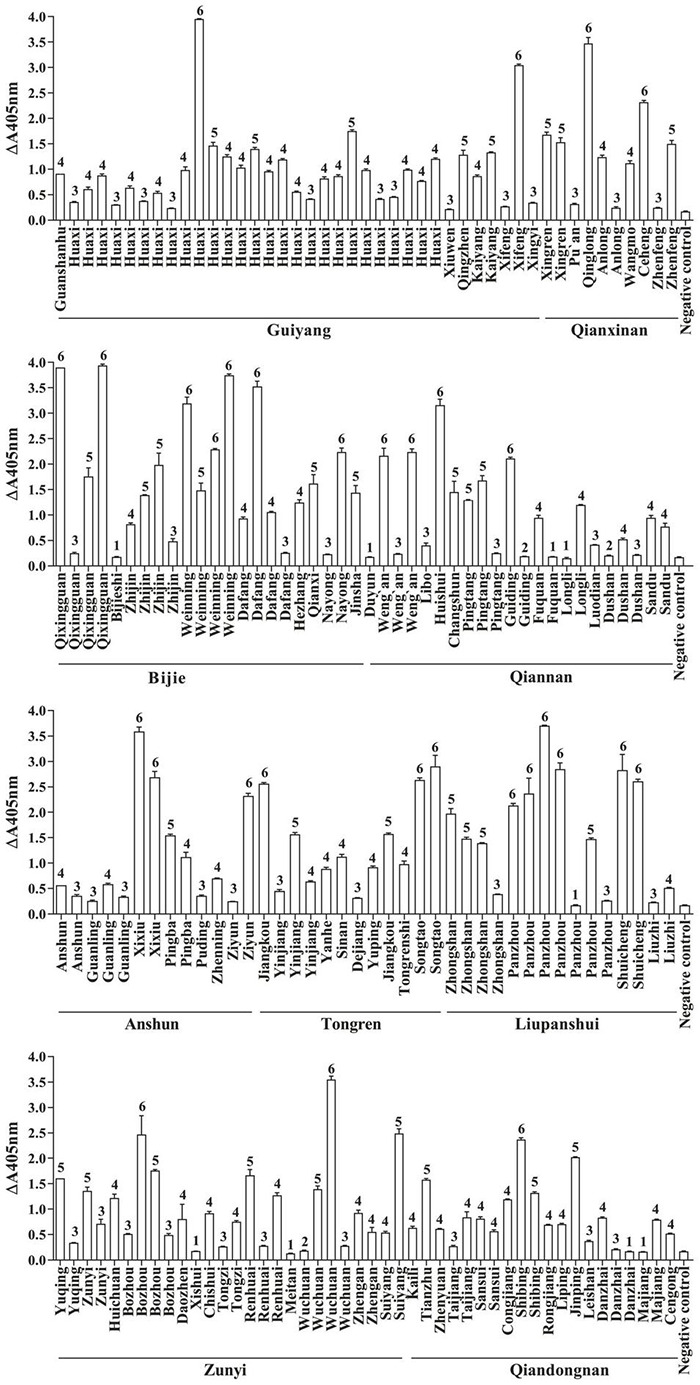
*Fusarium* infections in maize samples detected by rapid immunoassay. 1: *n* < 1.5, samples without *Fusarium* infections; 2: 1.5 ≤ *n* < 2, suspected infection samples; 3: 2 ≤ *n* < 10, mild infection samples; 4: 10 ≤ *n* < 30, moderate infection samples; 5: 30 ≤ *n* < 50, severe infection samples; 6: *n* ≥ 50, extreme infection samples; *n* = (OD_405 nm_ value of sample–OD_405 nm_ value of blank control)/(OD_405 nm_ value of negative control–OD_405 nm_ value of blank control).

FvSG7-AP fusions were induced for expression based on the optimization conditions, and a considerable quantity of soluble protein with high activity was obtained. A total of 175 diseased maize ears with similar symptoms, including kernels covered with white, pink or salmon-colored mold, were collected from 76 counties in Guizhou Province during the maize harvesting period. These kernels were pretreated and detected by rapid immunoassay using FvSG7-AP fusion proteins. As shown in Figure 1, only 5.14% (9/175) of samples displayed no difference compared to controls, 24% (42/175) had mild infections, 33.14% (58/175) had moderate infections, 18.86% (33/175) had serious infections, and 17.14% (30/175) had severe infections. In addition, 1.71% (3/175) of samples showed a small color reaction and were considered suspected of infections. Taken together, the incidence of *Fusarium* infections on collected maize samples was as high as 93.14%.

### Geographic Distribution of *Fusarium*-Infected Samples

**FIGURE 2:**
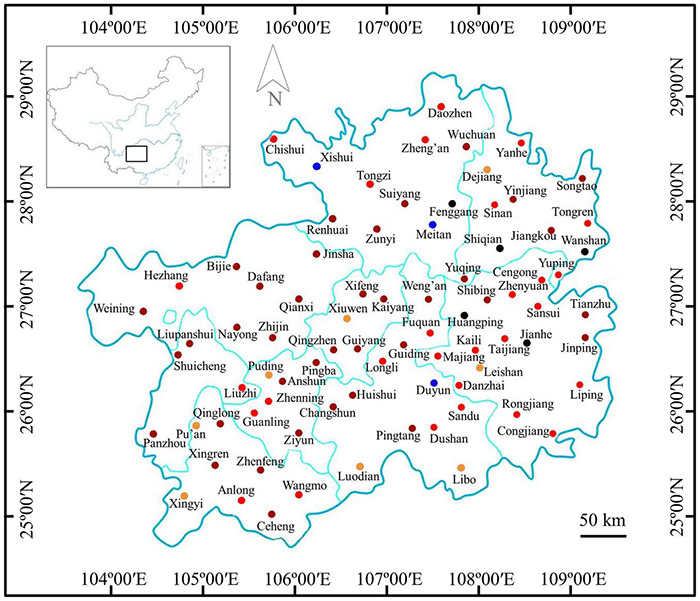
Distribution of *Fusarium*-infected samples in the Guizhou Province of China. 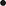: no maize sample collected; 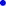: samples without *Fusarium* infections in these regions; 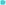: samples with suspected infections in these regions; 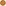: samples with mild infections in these regions; 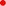 samples with moderate infections in these regions; and 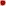: samples with severe and extreme infections in these regions.

The maize samples with or without *Fusarium* infections were marked on the map of Guizhou Province according to the results of immunoassay detection (Figure 2). The results showed that mild infected samples were detected in 10.53% (8/76) of regions, moderate infected samples were detected in 36.84% (28/76) of regions, severe and extreme infected samples were detected in 48.68% (37/76) of regions, and samples with *Fusarium* infections were not detected in 3.95% (3/76) of regions. Therefore, maize samples infected by *Fusarium* were found in 96.05% of counties of Guizhou Province, indicating a very wide distribution of *Fusarium* infections (Figure 2).

### Isolation and Molecular Identification of *Fusarium* Pathogens

**FIGURE 3:**
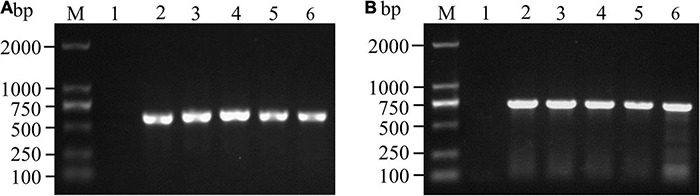
PCR amplification and agarose gel detection of *ITS* **(A)** and *TEF-1*α **(B)** genes. M: DNA molecular weight standard; Lane 1: negative control; Lanes A2-6: PCR products of the *ITS* gene of some isolates; Lanes B2-6: PCR products of the *TEF-1*α gene of some isolates.

The 163 samples that tested positive by immunoassay were biocultured, isolated and purified based on morphological characteristics (Nelson et al., 1983; Leslie and Summerell, 2006), and 139 isolates were tentatively identified as *Fusarium* species. The *ITS* and *TEF-1*α sequences were amplified, and agarose gel electrophoresis was performed to confirm these isolates. As shown in Figure 3, the *ITS* sequence was approximately 550 bp, while the fragment length of the *TEF-1*α gene was approximately 650 bp. BLSATn searches of sequence similarity identified 123 *Fusarium* isolates with an areal distribution rate of 82.19% (60/73).

### Population Structure Analysis of *Fusarium* Pathogens

**FIGURE 4:**
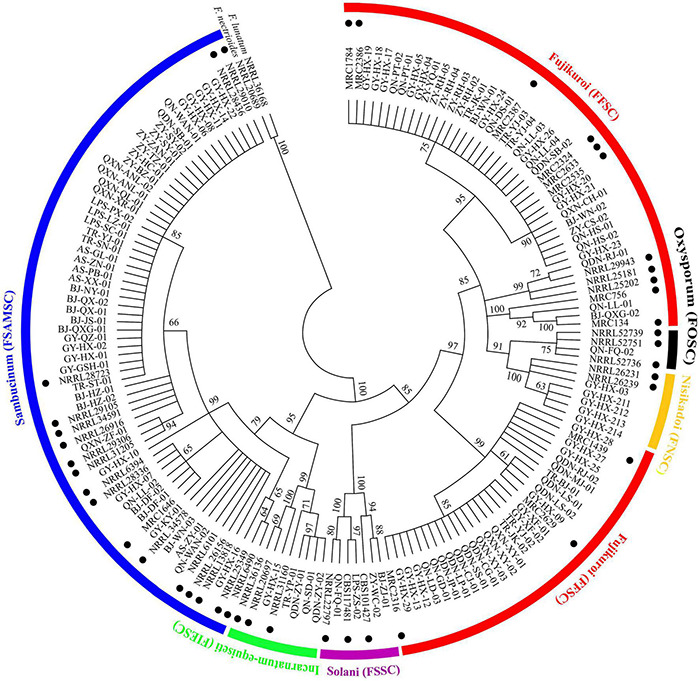
Phylogenetic tree of 123 *Fusarium* isolates based on the *TEF-1*α gene. Bootstrap values (≥60%) of a bootstrap test of 1,000 replicates are shown on branching nodes. Reference *TEF-1*α sequences of the NRRL/MRC/CBS strains were downloaded from the FUSARIUM-ID database and GenBank database. Sequences of *Fusarium nectrioides* and *Fusarium lunatum* were used as outgroups.

**FIGURE 5:**
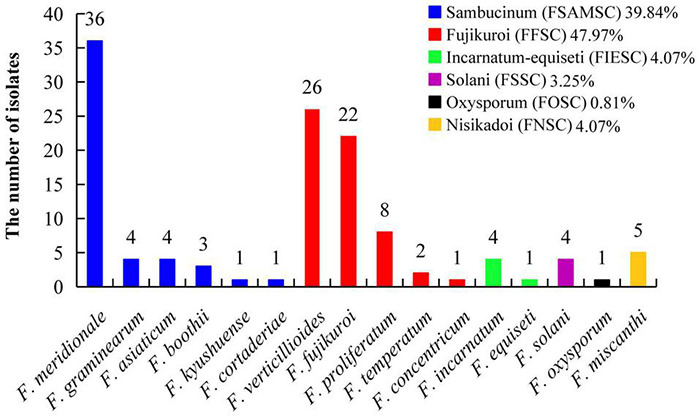
Classification of 123 *Fusarium* isolates based on sequence analysis of the *TEF-1α* gene.

A total of 123 *TEF-1*α sequences of *Fusarium* pathogens were successfully sequenced. The nucleotide sequences have been deposited in the GenBank database and the assigned accession numbers were listed in Supplementary Table 3. These sequences were aligned with MEGA-X-10.0.5 software, and then, the phylogenetic tree was constructed by using the maximum-likelihood method (Figure 4). As shown in Figure 5, 16 *Fusarium* species were characterized, including *Fusarium meridionale* (29.27%), *Fusarium verticillioides* (21.14%), *Fusarium fujikuroi* (17.89%), *Fusarium proliferatum* (6.50%), *Fusarium graminearum* (3.25%), *F. miscanthi* (4.07%), *Fusarium solani* (3.25%), *Fusarium incarnatum* (3.25%), *Fusarium asiaticum* (3.25%), *Fusarium temperatum* (1.63%), *Fusarium boothii* (2.44%), *F. concentricum* (0.81%), *Fusarium oxysporum* (0.81%), *Fusarium kyushuense* (0.81%), *F. cortaderiae* (0.81%), and *Fusarium equiseti* (0.81%). The 16 *Fusarium* species belonged to six species complexes, including the *Fusarium sambucinum* species complex (FSAMSC), *F. fujikuroi* species complex (FFSC), *Fusarium incarnatum-equiseti* species complex (FIESC), *F. solani* species complex (FSSC), *F. oxysporum* species complex (FOSC) and *Fusarium nisikadoi* species complex (FNSC) (Figures 4 and 5). At the same time, *F. meridionale* was identified as the dominant species causing maize ear rot in Guizhou Province, China. Moreover, four species, *F. kyushuense*, *F. concentricum*, *F. miscanthi*, and *F. cortaderiae* were first isolated from diseased maize samples in this area.

### Molecular Identification of Toxigenic Chemotypes

The genes involved in NIV, ZEN, DON, and FBs synthesis were detected by PCR amplification, and DNA fragments with the expected size were amplified from 56.10% (69/123) of isolates (Table 2). Among them, 31 isolates of *F. meridionale* and four isolates of *F. asiaticum* had NIV and ZEN genotypes; 34 isolates of *F. meridionale* had NIV or NIV+ZEN genotype; three isolates of *F. graminearum*, three isolates of *F. boothii* and one isolate of *F. cortaderiae* had DON+ZEN genotypes; 22 isolates of *F. verticillioides* and two isolates of *F. fujikuroi* had FBs genotype. The genes involved in DON synthesis were all detected from the *F. graminearum* species complex (FGSC), such as *F. graminearum*, *F. boothii*, and *F. cortaderiae*. The key genes for the synthesis of DON and ZEN were detected in *F. cortaderiae*, while the genes related to the synthesis of the four mycotoxins were not detected in *F. kyushuense*, *F. miscanthi* and *F. concentricum*.

**TABLE 2:** The chemotypes of 123 *Fusarium* isolates identified by PCR amplification.

Species	Number of *Fusarium* isolates	Chemotypes			
		NIV	NIV + ZEN	DON + ZEN	FBs
*F. meridionale*	36	3	31	0	0
*F. graminearum*	4	0	0	3	0
*F. asiaticum*	4	0	4	0	0
*F. boothii*	3	0	0	3	0
*F. cortaderiae*	1	0	0	1	0
*F. kyushuense*	1	0	0	0	0
*F. verticillioides*	26	0	0	0	22
*F. fujikuroi*	22	0	0	0	2
*F. proliferatum*	8	0	0	0	0
*F. temperatum*	2	0	0	0	0
*F. concentricum*	1	0	0	0	0
*F. incarnatum*	4	0	0	0	0
*F. equiseti*	1	0	0	0	0
*F. solani*	4	0	0	0	0
*F. oxysporum*	1	0	0	0	0
*F. miscanthi*	5	0	0	0	0
TOTAL	123	3	35	7	24

**FIGURE 6:**
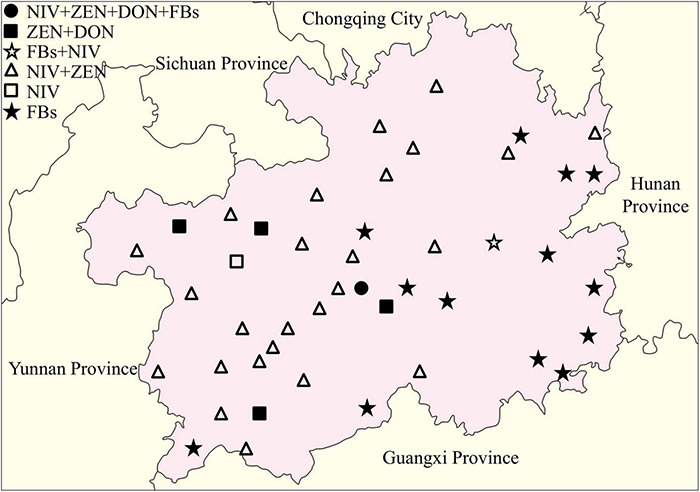
Distribution of toxigenic genotypes of *Fusarium* isolates in the Guizhou Province of China.

The distribution of toxigenic chemotypes of the *Fusarium* isolates showed that the *Fusarium* isolates with the potential to produce both NIV and ZEN were largely distributed in western and northern Guizhou Province, whereas the *Fusarium* isolates with FBs genotype were mainly concentrated in southeastern Guizhou (Figure 6). Eleven representative isolates were cultured, pretreated and then subjected to LC-MS/MS detection for the confirmation of mycotoxin chemtypes. The results of the representative isolates were generally consistent with the PCR results. After inoculated with two *F. verticillioides* isolates, the contents of FB_1_, FB_2_, and FB_3_ in the cultures reached 270.7281–2269.3430 μg/g, 117.1875–1326.5330 μg/g, and 143.0563–1038.0720 μg/g, respectively. Intriguingly, FB_1_ and FB_2_ ranging from 0.0854 to 0.3653 μg/g were measured in the medium that separately inoculated the isolates of *F. miscanthi* and *F. concentricum* (Supplementary Table 4).

## Discussion

*Fusarium* pathogens show rapid mycelial growth under hot and humid environments, but the optimal temperature for toxin production is 15∼25°C (Samapundo et al., 2005; Rybecky et al., 2018). Thus, *Fusarium* infection of crops is often asymptomatic with high levels of mycotoxin production (Munkvold and Desjardins, 2007). Guizhou is located on the Yunnan-Kweichow Plateau, which has a plateau humid subtropical monsoon climate with the characteristics of high humidity, low temperature, and diversity. Theoretically, this is not satisfactory for the rapid growth of hyphae, but it may be suitable for the production of mycotoxins. At present, the methods applied to monitor *Fusarium* are mainly biological characterization and molecular detection. However, these methods require the cultivation of fungi before detection, and the sample pretreatment process is complicated, time-consuming and laborious. Remarkably, immunoassays not only have high specificity and sensitivity but are also adaptable to high-throughput screening and simple sample preparation. The prokaryotic expression of scFv-AP fusion proteins by genetic engineering technology is considered a good choice for rapid immunological detection (Hu et al., 2013). Based on the formerly constructed scFv-AP fusion and optimal expression conditions (Hu et al., 2013; Wang et al., 2015), this study completed large-scale expression of the FvSG7-AP fusion protein for detection of *Fusarium* infections. A rapid immunoassay was deployed to measure 175 rotted maize ear samples collected from 76 counties in Guizhou Province, China. The results showed that the incidence rate of *Fusarium* infections in these samples reached 93.14% and the distribution was widespread (Figure 2). Therefore, the status of *Fusarium* infections in this area needs to be given sufficient attention.

Given that accurate classification of *Fusarium* species is the basis for further monitoring and controlling of pathogen infections and mycotoxin contamination, morphological identification and molecular phylogenetic analysis were applied to identify the collected *Fusarium* isolates. Traditional morphological identification has difficulty accurately distinguishing *Fusarium* isolates at the species level. Phylogenetic analysis of DNA sequences has been widely used to assess the genetic relationship of *Fusarium* species (Wang et al., 2014b). The *TEF-1*α gene provides much better identification among and within lineages than other loci, such as the β*-TUB*, *CAM* and *ITS* regions. In addition, there are no orthologous copies of *TEF-1*α in this genus, making this locus a better candidate for distinguishing phylogenetic relationships (Amarasinghe et al., 2019). Therefore, classification and identification of *Fusarium* species based on *TEF-1*α gene sequences has become the most common method (Mirete et al., 2004; Hafez et al., 2020). Geiser et al. (2004) established a *Fusarium* database based on the partial sequence of the *TEF-1*α gene, which allows researchers to easily identify species under *Fusarium* spp. based on DNA sequencing results. In this study, the subculture and purification of *Fusarium* pathogens from 163 positive samples were performed according to the results of immunoassays, making the performance more accurate and efficient. According to morphological characteristics (Nelson et al., 1983; Leslie and Summerell, 2006), 139 isolates were initially identified. Further molecular identification by *ITS* and *TEF-1*α sequences showed that 123 isolates were characterized as *Fusarium* spp. at the species level. The *Fusarium* spp. were not successfully characterized in 40 samples, which might due to the competitive inhibition of other fungi and bacteria in biological culture and inaccurate morphological identification. The nucleotide sequences of the GY-HX-10 isolate had the highest identity (98.3%) with the sequence of *F. boothii* NRRL 29105 (GenBank accession no. AF212446), and it was separated as a single branch. Further identification using the RPB2 gene confirmed its high identity (99.8%) with the sequence of *F. boothii* NRRL 26916 (GenBank accession no. GQ915487). In this study, *F. kyushuense*, *F. concentricum*, *F. miscanthi*, and *F. cortaderiae* were first isolated from rotted maize ears in the Guizhou area. *F. kyushuense* was first isolated from diseased wheat in Japan (Aoki and O’Donnell, 1998), and first reported to cause tobacco wilt in the Guizhou Province of China (Wang H. C. et al., 2013) and cause maize ear rot in the Anhui, Hubei, and Yunnan provinces of China (Wang et al., 2014a). *F. concentricum* was first reported to cause pepper fruit rot in China (Wang J. H. et al., 2013) and cause maize ear rot in the Guangxi Zhuang Autonomous Region of China (Du et al., 2020). *F. miscanthi* was first isolated from the straw of Japanese silver grass, *Miscanthus* sinensis, in Denmark (Gams et al., 1999) and identified to be infectious agent of maize ear rot (Shang et al., 2021). *F. cortaderiae* was first isolated from pampas grass (*Cortaderia jubata*) (O’Donnell et al., 2004) and subsequently separated from maize grain in New Zealand (Monds et al., 2005). *F. cortaderiae* was first reported to cause head blight on annual ryegrass in Brazil (Machado et al., 2015) and found to be the causal agent of maize stalk rot disease in the Yunnan Province of China (Xi et al., 2021).

According to the classification of *Fusarium* by O’Donnell et al. (2013, 2015), FSAMSC covers approximately 50 different species, including FGSC. FGSC is considered to be a worldwide population consisting of at least 16 phylogenetic species, some of which have a specific geographical distribution (Chiotta et al., 2016; Hao et al., 2017). In this study, six different phylogenetic FSAMSCs were classified, of which 97.96% belonged to FGSC (*F. graminearum*, *F. meridionale*, *F. boothii*, *F. asiaticum*, and *F. cortaderiae*). This is consistent with the results that maize ear rot in northern China was mainly caused by *F. verticillioides* and that, in the southern area, it was primarily infected by a compound species of FGSC (Qin et al., 2014). This study showed that the dominant pathogen of maize ear rot in Guizhou Province is *F. meridionale*, followed by *F. verticillioides* and *F. fujikuroi*. Zhou et al. (2018) reported that *F. verticillioides*, *F. proliferatum*, and *F. meridionale* were the predominant fungi causing maize ear and kernel rot in Chongqing City, China. Fu et al. (2015) reported that *F. graminearum* was the dominant species in Yunnan, Guizhou and Shanxi Provinces. However, Xi et al. (2021) reported that *F. meridionale* was the dominant pathogen of maize stalks in Yunnan Province, China, and *F. graminearum* was only 0.5%. This difference may be related to the specific geographical environment and annual climate differences.

Nivalenol, ZEN, DON, and FBs are the major mycotoxin contaminants caused by *Fusarium* species (Zhou et al., 2018). This study applied PCR amplification to identify the chemotypes of isolates based on specific genes involved in NIV, ZEN, DON and FBs synthesis. A total of 69 isolates were identified as potential mycotoxin-producing species, of which 55.07% (38/69) were NIV and 60.87% (42/69) were the ZEN chemotype because the dominant species in the region was *F. meridionale*. Previous studies have reported that *F. meridionale* isolated from soybean in *Argentina* can simultaneously produce DON and NIV toxins (Rybecky et al., 2018), whereas *F. meridionale* isolated from maize ear rot in *Argentina* only produces NIV (Sampietro et al., 2011). This study found that both NIV and ZEN synthesis-related genes were amplified from 86.11% (31/36) of *F. meridionale*, but the *Tri5–Tri6* gene for DON synthesis was not detected. The description of mycotoxin chemotypes may be associated with many factors, such as the host and environmental conditions. According to other reports, the optimal conditions for *F. meridionale* growth were 25°C and high water activity, which is also suitable for toxin production (Rybecky et al., 2018; Belizan et al., 2019). The climatic characteristics of warm, humid and abundant rainfall during the maize-harvesting period (August to October) in Guizhou Province may be one of the reasons for serious *Fusarium* infection and mycotoxin accumulation. Fumonisin synthesis-related genes of *FUM* are located in the same gene family. In the biosynthetic pathway of fumonisins, some of the products of *FUM* genes play a major catalytic role, and the rest play indirect roles (Alexander et al., 2009). The *FUM* genes were mostly detected in the isolates of *F. verticillioides*, which is consistent with previous studies (Duan et al., 2016; Zhou et al., 2018). Unusually, the *FUM* genes were not amplified from the isolates of *F. proliferatum* and *F. temperatum*. Although *F. proliferatum* is one of the main FBs-producing species and verified to be completely synthetic with the fumonisin gene cluster of *F. verticillioides* (Sun et al., 2019), some studies have shown that the deletion of *FUM1* gene can reduce FB_1_ production by 99% (Duan et al., 2016). At the same time, a PCR analysis of 20 *F. proliferatum* isolates by using *FUM1* gene-specific primers has been conducted and the gene fragments were obtained only from thirteen isolates (Dissanayake et al., 2009). Scauflaire et al. (2011) first discovered *F. temperatum* in Belgium and identified it as a new species, but only one of the eleven *F. temperatum* species produced a small amount of FB_1_ (Scauflaire et al., 2012). Fumero et al. (2020) conducted chemical and genotype identification of 12 *F. temperatum* isolates from Argentina and showed that neither fumonisin production nor *FUM* biosynthesis genes were detected. Wang et al. (2014b) reported that 10 isolates of *F. temperatum* isolated in Zunyi and Guiyang of Guizhou Province can produce FB_1_ and FB_2_. The differences in the mycotoxin spectrum may reflect the genetic variation between *F. temperatum* isolates in different geographic regions.

Although the pretreatment process is complex, LC–MS/MS is widely used for the accurate determination of mycotoxin chemotypes (Duan et al., 2016). In this study, some isolates were randomly selected to culture and measure their mycotoxin production by LC–MS/MS. The results confirmed the molecular identification, suggesting that PCR amplification is a rapid and effective method. Also, our result showed that *F. verticillioides* has the potential to produce high levels of FBs and is one of the dominant species of maize ear rot pathogens in this area. Therefore, it is necessary to strengthen the monitoring of FBs contamination in food in this area. In addition, a certain amount of FBs was detected in the cultures of *F. miscanthi* and *F. concentricum* isolates, from which the *FUM* gene fragment was not amplified. Certainly, the content of FBs was far lower than that produced by the molecularly identified isolates, which may be correlated with the expression and regulation of toxin synthesis-related genes (Zhou et al., 2018). This study is the first report that *F. miscanthi* and *F. concentricum* can produce FBs.

## Conclusion

This study combined immunoassays and molecular identification for the first time to systematically investigate maize ear rot caused by *Fusarium* pathogens as well as their mycotoxin chemotypes in Guizhou Province, China. The results displayed a variety of *Fusarium* species distributed almost all over the whole area, and *F. meridionale* was the predominant species. *F. kyushuense*, *F. concentricum*, *F. miscanthi*, and *F. cortaderiae* were first isolated from maize ear rot in the Guizhou area. Further molecular identification and LC–MS/MS confirmation showed that most of these *Fusarium* isolates have the potential to produce mycotoxins with typical geographical distribution features. Together, scFv-AP fusion-based immunoassay is proven an effective method to detect fungal infections in crops, and more attention and measures should be taken to ensure human and animal health in Guizhou Province.

## Data Availability Statement

The data presented in the study are deposited in the GenBank repository, accession number of 123 genes from 123 Fusarium isolates are listed in Supplementary Table 3.

## Author Contributions

ZH, JZ, and ZZ: conceptualization, project administration, and funding acquisition. ZH, JZ, and GS: methodology. GS and SQL: data curation. GS and YW: software. GS, SQL, and HY: formal analysis. GS, SML, JY, HY, YY, and JW: investigation. JY, HY, SML, YY, and JW: validation. ZH, ZZ, JZ, and YW: resources and writing-review and editing. GS, SQL, and HY: writing—original draft preparation. ZH and YW: supervision. All authors have read and agreed to the published version of the manuscript.

## Conflict of Interest

The authors declare that the research was conducted in the absence of any commercial or financial relationships that could be construed as a potential conflict of interest.

## Publisher’s Note

All claims expressed in this article are solely those of the authors and do not necessarily represent those of their affiliated organizations, or those of the publisher, the editors and the reviewers. Any product that may be evaluated in this article, or claim that may be made by its manufacturer, is not guaranteed or endorsed by the publisher.

## Footnotes

1
http://www.dnastar.com/

2
http://isolate.fusariumdb.org/guide.php

3
http://www.ncbi.nlm.nih.gov/
