# Supplementary material for: An Efficient Strategy Combining Immunoassays and Molecular Identification for the Investigation of Fusarium Infections in Ear Rot of Maize in Guizhou Province, China
Source: Front Microbiol. 2022 Mar 14;13:849698. doi: 10.3389/fmicb.2022.849698 (PMC8964309; doi:10.3389/fmicb.2022.849698)
Supplement: Supplementary file 1 [file Table_1.DOCX]

**Supplementary TABLE 1 | The information of reference strains and their GenBank accession numbers.**

| **Strain number^a^** | **Species** | **Complex^b^** | **Accession number** |
| --- | --- | --- | --- |
| NRRL 29010 | *F. meridionale* | FSAMSC | AF212437 |
| NRRL 28436 | *F. meridionale* | FSAMSC | AF212435 |
| NRRL 28723 | *F. meridionale* | FSAMSC | AF212436 |
| NRRL 29105 | *F. boothii* | FSAMSC | AF212446 |
| NRRL 34591 | *F. boothii* | FSAMSC | EF428715 |
| NRRL 26916 | *F. boothii* | FSAMSC | GQ915503 |
| NRRL 29306 | *F. cortaderiae* | FSAMSC | AY225886 |
| NRRL 31205 | *F. cortaderiae* | FSAMSC | AY452960 |
| NRRL 6394 | *F. graminearum* | FSAMSC | AF212456 |
| NRRL 28336 | *F. graminearum* | FSAMSC | AF212459 |
| MRC 1646 | *F. graminearum* | FSAMSC | MH582246 |
| NRRL 6101 | *F. asiaticum* | FSAMSC | AF212450 |
| NRRL 13818 | *F. asiaticum* | FSAMSC | MW233069 |
| NRRL 34578 | *F. asiaticum* | FSAMSC | EF428710. |
| NRRL 26156 | *F. asiaticum* | FSAMSC | AF212452 |
| NRRL 25349 | *F. kyushuense* | FSAMSC | GQ915508 |
| NRRL 6490 | *F. kyushuense* | FSAMSC | AB674297 |
| NRRL 36136 | *F. equiseti* | FIESC | GQ505644 |
| NRRL 20697 | *F. equiseti* | FIESC | GQ505594 |
| NRRL 31160 | *F. incarnatum* | FIESC | GQ915510 |
| NRRL 22797 | *F. solani* | FSSC | DQ247667 |
| CBS 117481 | *F. solani* | FSSC | HE647957 |
| CBS 101427 | *F. solani* | FSSC | DQ246834 |
| MRC 2316 | *F. verticillioides* | FFSC | MH582317 |
| MRC 1439 | *F. verticillioides* | FFSC | MH582325 |
| MRC 2629 | *F. verticillioides* | FFSC | MH582328 |
| NRRL 26239 | *F. miscanthi* | FNSC | AF324332 |
| NRRL 26231 | *F. miscanthi* | FNSC | AF324331 |
| NRRL 52736 | *F. oxysporum* | FOSC | JF740813 |
| NRRL 52751 | *F. oxysporum* | FOSC | JF740826 |
| NRRL 52739 | *F. oxysporum* | FOSC | JF740816 |
| MRC 134 | *F. temperatum* | FFSC | MH582312 |
| MRC 756 | *F. temperatum* | FFSC | MH582313 |
| NRRL 29943 | *F. concentricum* | FFSC | AF333934 |
| NRRL 25181 | *F. concentricum* | FFSC | AF160282 |
| NRRL 25202 | *F. concentricum* | FFSC | JF740760 |
| MRC 2535 | *F. proliferatum* | FFSC | MH582346 |
| MRC 2633 | *F. proliferatum* | FFSC | MH582345 |
| MRC 2324 | *F. proliferatum* | FFSC | MH582344 |

*(Continued)*

**Supplementary TABLE 1 | Continued**

| **Strain number^a^** | **Species** | **Complex^b^** | **Accession number** |
| --- | --- | --- | --- |
| MRC 2387 | *F. fujikuroi* | FFSC | MH582340 |
| MRC 2386 | *F. fujikuroi* | FFSC | MH582339 |
| MRC 1784 | *F. fujikuroi* | FFSC | MH582338 |
| NRRL 20689 | *F. nectrioides* | FDSC | EU926312 |
| NRRL 36168 | *F. lunatum* | FDSC | EU926291 |

**^a^** Strain number: Tester reference strains are. **CBS**, Centraalbureau voor Schimmelcultures, Utrecht, the Netherlands; **MRC**, Medical Research Council, Tygerberg, South Africa; **NRRL**, Northern Regional Research Laboratory, NCAUR, Peoria, Illinois. **^b^ FSAMSC**, *Fusarium sambucinum* species complex; **FIESC**, *Fusarium incarnatum-equiseti* species complex; **FSSC**, *Fusarium solani* Fusarium; **FFSC**, *Fusarium fujikuroi* species complex; **FNSC**, *Fusarium nisikadoi* species complex; **FOSC**, *Fusarium oxysporum* species complex; **FDSC**, *Fusarium* *dimerum* species complex;
