# Supplementary material for: An Efficient Strategy Combining Immunoassays and Molecular Identification for the Investigation of Fusarium Infections in Ear Rot of Maize in Guizhou Province, China
Source: Front Microbiol. 2022 Mar 14;13:849698. doi: 10.3389/fmicb.2022.849698 (PMC8964309; doi:10.3389/fmicb.2022.849698)
Supplement: Supplementary file 2 [file Table_2.DOCX]

**Supplementary TABLE 2 | The specific MS parameters for six target in MS/MS analysis.**

| **Analytes** | **Molecular**  **weight (g/mol)** | **T_R_ (min)** | **Molecular**  **ion** | **ESI** | **Parent**  **ions (*m/z*)** | **Product ions (*m/z*)** | **CE (eV)** |
| --- | --- | --- | --- | --- | --- | --- | --- |
| DON | 296.32 | 5.08 | [M+H]^+^ | ES^+^ | 297.10 | 249.20^a^ | 16.000 |
|  |  |  |  |  | 297.10 | 203.00 | 22.000 |
| NIV | 312.32 | 3.78 | [M-H]^-^ | ESI^-^ | 310.90 | 281.00^a^ | -14.820 |
|  |  |  |  |  | 310.90 | 205.00 | -23.940 |
| ZEN | 318.36 | 10.6 | [M-H]^-^ | ESI^-^ | 317.20 | 175.00^a^ | -33.500 |
|  |  |  |  |  | 317.20 | 273.00 | -27.700 |
| FB_1_ | 721.83 | 9.00 | [M+H]^+^ | ES^+^ | 722.40 | 352.40^a^ | 53.500 |
|  |  |  |  |  | 722.40 | 334.30 | 53.500 |
| FB_2_ | 705.83 | 12.7 | [M+H]^+^ | ES^+^ | 706.00 | 336.00^a^ | 45.000 |
|  |  |  |  |  | 706.00 | 354.00 | 35.000 |
| FB_3_ | 705.83 | 10.4 | [M+H]^+^ | ES^+^ | 706.00 | 336.00^a^ | 45.000 |
|  |  |  |  |  | 706.00 | 354.00 | 35.000 |

^a^ Quantitative ion
