# Supplementary material for: An Efficient Strategy Combining Immunoassays and Molecular Identification for the Investigation of Fusarium Infections in Ear Rot of Maize in Guizhou Province, China
Source: Front Microbiol. 2022 Mar 14;13:849698. doi: 10.3389/fmicb.2022.849698 (PMC8964309; doi:10.3389/fmicb.2022.849698)
Supplement: Supplementary file 3 [file Table_3.DOCX]

**Supplementary TABLE 3 | The information and GenBank accession numbers of *Fusarium* isolates identified in this study.**

| **Isolate** | **Locations** | **Species** | **Accession number** |
| --- | --- | --- | --- |
| BJ-DF-01 | Dafang, Bijie | *F.graminearum* | OM103429 |
| GY-HX-07 | Huaxi, Guiyang | *F.graminearum* | OM103430 |
| QN-LL-02 | Longli, Qiannan | *F.graminearum* | OM103431 |
| BJ-DF-02 | Dafang, Bijie | *F.graminearum* | OM103432 |
| GY-KY-01 | Kaiyang, Guiyang | *F. asiaticum* | OM103433 |
| BJ-WN-03 | Weining, Bijie | *F. asiaticum* | OM103434 |
| AS-ZY-01 | Ziyun, Anshun | *F. asiaticum* | OM103435 |
| QN-W`AN-02 | Weng`an, Qiannan | *F. asiaticum* | OM103436 |
| BJ-HZ-01 | Hezhang, Bijie | *F. boothii* | OM103437 |
| BJ-HZ-02 | Hezhang, Bijie | *F. boothii* | OM103438 |
| GY-HX-10 | Huaxi, Guiyang | *F. boothii* | OM103439 |
| QXN-ZF-01 | Zhenfeng, Qianxinan | *F. cortaderiae* | OM103440 |
| GY-HX-15 | Huaxi, Guiyang | *F. equiseti* | OM103441 |
| GY-HX-16 | Huaxi, Guiyang | *F. kyushuense* | OM103442 |
| QN-FQ-02 | Fuquan, Qiannan | *F. oxysporum* | OM103443 |
| BJ-QXG-02 | Qixingguan, Bijie | *F. temperatum* | OM103444 |
| QN-LL-01 | Longli, Qiannan | *F. temperatum* | OM103445 |
| GY-XF-01 | Xifeng, Guiyang | *F. verticillioides* | OM103446 |
| GY-XF-02 | Xifeng, Guiyang | *F. verticillioides* | OM103447 |
| TR-BJ-01 | Bijiang, Tongren | *F. verticillioides* | OM103448 |
| TR-YJ-02 | Yinjiang, Tongren | *F. verticillioides* | OM103449 |
| TR-JK-02 | Jiangkou, Tongren | *F. verticillioides* | OM103450 |
| QXN-XY-01 | Xingyi, Qianxinan | *F. verticillioides* | OM103451 |
| QXN-XY-02 | Xingyi, Qianxinan | *F. verticillioides* | OM103452 |
| QXN-XY-03 | Xingyi, Qianxinan | *F. verticillioides* | OM103453 |
| QDN-CG-01 | Cengong, Qiandongnan | *F. verticillioides* | OM103454 |
| QDN-SS-01 | Sansui, Qiandongnan | *F. verticillioides* | OM103455 |
| QDN-MJ-01 | Majiang, Qiandongnan | *F. verticillioides* | OM103456 |
| QDN-RJ-02 | Rongjiang, Qiandongnan | *F. verticillioides* | OM103457 |
| QDN-CJ-01 | Congjiang, Qiandongnan | *F. verticillioides* | OM103458 |
| QDN-LP-01 | Liping, Qiandongnan | *F. verticillioides* | OM103459 |
| QDN-JP-01 | Jinping, Qiandongnan | *F. verticillioides* | OM103460 |
| QDN-LS-01 | Leishan, Qiandongnan | *F. verticillioides* | OM103461 |
| QDN-LS-02 | Leishan, Qiandongnan | *F. verticillioides* | OM103462 |
| QN-GD-01 | Guiding, Qiannan | *F. verticillioides* | OM103463 |
| QN-LD-03 | Luodian, Qiannan | *F. verticillioides* | OM103464 |
| GY-HX-09 | Huaxi, Guiyang | *F. verticillioides* | OM103465 |
| GY-HX-12 | Huaxi, Guiyang | *F. verticillioides* | OM103466 |

*(Continued)*

**Supplementary Table 3 | Continued**

| **Isolate** | **Locations** | **Species** | **Accession number** |
| --- | --- | --- | --- |
| GY-HX-13 | Huaxi, Guiyang | *F. verticillioides* | OM103467 |
| GY-HX-25 | Huaxi, Guiyang | *F. verticillioides* | OM103468 |
| GY-HX-27 | Huaxi, Guiyang | *F. verticillioides* | OM103469 |
| GY-HX-28 | Huaxi, Guiyang | *F. verticillioides* | OM103470 |
| GY-HX-29 | Huaxi, Guiyang | *F. verticillioides* | OM103471 |
| GY-GSH-01 | Guanshanhu, Guiyang | *F. meridionale* | OM103472 |
| GY-HX-01 | Huaxi, Guiyang | *F. meridionale* | OM103473 |
| GY-HX-02 | Huaxi, Guiyang | *F. meridionale* | OM103474 |
| GY-QZ-01 | Qingzhen, Guiyang | *F. meridionale* | OM103475 |
| BJ-QXG-01 | Qixingguan, Bijie | *F. meridionale* | OM103476 |
| BJ-JS-01 | Jinsha, Bijie | *F. meridionale* | OM103477 |
| BJ-QX-01 | Qianxi, Bijie | *F. meridionale* | OM103478 |
| BJ-QX-02 | Qianxi, Bijie | *F. meridionale* | OM103479 |
| BJ-NY-01 | Nayong, Bijie | *F. meridionale* | OM103480 |
| AS-XX-01 | Xixiu, Anshun | *F. meridionale* | OM103481 |
| AS-PB-01 | Pingba, Anshun | *F. meridionale* | OM103482 |
| AS-ZN-01 | Zhenning, Anshun | *F. meridionale* | OM103483 |
| AS-ZN-01 | Zhenning, Anshun | *F. meridionale* | OM103484 |
| TR-SN-01 | Sinan, Tongren | *F. meridionale* | OM103485 |
| TR-YJ-01 | Yinjiang, Tongren | *F. meridionale* | OM103486 |
| TR-ST-01 | Songtao, Tongren | *F. meridionale* | OM103487 |
| LPS-SC-01 | Shuicheng, Liupanshui | *F. meridionale* | OM103488 |
| LPS-LZ-01 | Liuzhi, Liupanshui | *F. meridionale* | OM103489 |
| LPS-PX-02 | Panzhou, Liupanshui | *F. meridionale* | OM103490 |
| QXN-XR-01 | Xingren, Qianxinan | *F. meridionale* | OM103491 |
| QXN-QL-01 | Qinglong, Qianxinan | *F. meridionale* | OM103492 |
| QXN-AN`L-01 | Anlong, Qianxinan | *F. meridionale* | OM103493 |
| QXN-AN`L-02 | Anlong, Qianxinan | *F. meridionale* | OM103494 |
| ZY-BZ-01 | Bozhou, Zunyi | *F. meridionale* | OM103495 |
| ZY-HC-01 | Huichuan, Zunyi | *F. meridionale* | OM103496 |
| ZY-TZ-01 | Tongzi, Zunyi | *F. meridionale* | OM103497 |
| ZY-Z`AN-01 | Zheng`an, Zunyi | *F. meridionale* | OM103498 |
| ZY-SY-01 | Suiyang, Zunyi | *F. meridionale* | OM103499 |
| ZY-SY-02 | Suiyang, Zunyi | *F. meridionale* | OM103500 |
| QDN-SB-01 | Shibing, Qiandongnan | *F. meridionale* | OM103501 |
| QN-W`AN-01 | Weng`an, Qiannan | *F. meridionale* | OM103502 |
| GY-HX-06 | Huaxi, Guiyang | *F. meridionale* | OM103503 |
| GY-HX-08 | Huaxi, Guiyang | *F. meridionale* | OM103504 |
| GY-HX-11 | Huaxi, Guiyang | *F. meridionale* | OM103505 |
| GY-HX-14 | Huaxi, Guiyang | *F. meridionale* | OM103506 |

*(Continued)*

**Supplementary Table 3 | Continued**

| **Isolate** | **Locations** | **Species** | **Accession mumber** |
| --- | --- | --- | --- |
| GY-HX-22 | Huaxi, Guiyang | *F. meridionale* | OM103507 |
| QN-SD-01 | Sandu, Qiannan | *F. incarnatum* | OM103508 |
| QDN-ZY-01 | Zhenyuan, Qiandongnan | *F. incarnatum* | OM103509 |
| QDN-ZY-02 | Zhenyuan, Qiandongnan | *F. incarnatum* | OM103510 |
| TR-YP-01 | Yuping, Tongren | *F. incarnatum* | OM103511 |
| BJ-WN-02 | Weining, Bijie | *F. proliferatum* | OM103512 |
| GY-HX-20 | Huaxi, Guiyang | *F. proliferatum* | OM103513 |
| GY-HX-21 | Huaxi, Guiyang | *F. proliferatum* | OM103514 |
| GY-HX-23 | Huaxi, Guiyang | *F. proliferatum* | OM103515 |
| QXN-CH-01 | Ceheng, Qianxinan | *F. proliferatum* | OM103516 |
| QN-HS-01 | Huishui, Qiannan | *F. proliferatum* | OM103517 |
| QN-HS-02 | Huishui, Qiannan | *F. proliferatum* | OM103518 |
| ZY-CS-02 | Chishui, Zunyi | *F. proliferatum* | OM103519 |
| BJ-WN-01 | Weining, Bijie | *F. fujikuroi* | OM103520 |
| GY-HX-04 | Huaxi, Guiyang | *F. fujikuroi* | OM103521 |
| GY-HX-17 | Huaxi, Guiyang | *F. fujikuroi* | OM103522 |
| GY-HX-24 | Huaxi, Guiyang | *F. fujikuroi* | OM103523 |
| QN-DS-01 | Dushan, Qiannan | *F. fujikuroi* | OM103524 |
| QDN-SB-02 | Shibing, Qiandongnan | *F. fujikuroi* | OM103525 |
| TR-YJ-03 | Yinjiang, Tongren | *F. fujikuroi* | OM103526 |
| TR-YJ-04 | Yinjiang, Tongren | *F. fujikuroi* | OM103527 |
| TR-JK-01 | Jiangkou, Tongren | *F. fujikuroi* | OM103528 |
| ZY-RH-02 | Renhuai, Zunyi | *F. fujikuroi* | OM103529 |
| ZY-YQ-01 | Yuqing, Zunyi | *F. fujikuroi* | OM103530 |
| QN-LL-03 | Longli, Qiannan | *F. fujikuroi* | OM103531 |
| GY-HX-05 | Huaxi, Guiyang | *F. fujikuroi* | OM103532 |
| GY-HX-18 | Huaxi, Guiyang | *F. fujikuroi* | OM103533 |
| GY-HX-19 | Huaxi, Guiyang | *F. fujikuroi* | OM103534 |
| QN-LL-04 | Longli, Qiannan | *F. fujikuroi* | OM103535 |
| ZY-RH-03 | Renhuai, Zunyi | *F. fujikuroi* | OM103536 |
| ZY-RH-04 | Renhuai, Zunyi | *F. fujikuroi* | OM103537 |
| ZY-RH-05 | Renhuai, Zunyi | *F. fujikuroi* | OM103538 |
| QN-PT-01 | Pingtang, Qiannan | *F. fujikuroi* | OM103539 |
| GY-HX-26 | Huaxi, Guiyang | *F. fujikuroi* | OM103540 |
| QN-PT-02 | Pingtang, Qiannan | *F. fujikuroi* | OM103541 |
| BJ-ZJ-01 | Zhijin, Bijie | *F. solani* | OM135576 |
| LPS-ZS-02 | Zhongshan, Liupanshui | *F. solani* | OM135577 |
| QN-FQ-01 | Fuquan, Qiannan | *F. solani* | OM135578 |
| ZY-WC-02 | Wuchuan, Zunyi | *F. solani* | OM135579 |
| GY-HX-211 | Huaxi, Guiyang | *F. miscanthi* | MN750830 |

*(Continued)*

**Supplementary Table 3 | Continued**

| **Isolate** | **Locations** | **Species** | **Accession number** |
| --- | --- | --- | --- |
| GY-HX-212 | Huaxi, Guiyang | *F. miscanthi* | MN750831 |
| GY-HX-213 | Huaxi, Guiyang | *F. miscanthi* | MN750832 |
| GY-HX-214 | Huaxi, Guiyang | *F. miscanthi* | MN750833 |
| GY-HX-03 | Huaxi, Guiyang | *F. miscanthi* | MN750829 |
| QN-RJ-01 | Rongjiang, Qiannan | *F. concentricum* | MK814880 |
