# Supplementary material for: An Efficient Strategy Combining Immunoassays and Molecular Identification for the Investigation of Fusarium Infections in Ear Rot of Maize in Guizhou Province, China
Source: Front Microbiol. 2022 Mar 14;13:849698. doi: 10.3389/fmicb.2022.849698 (PMC8964309; doi:10.3389/fmicb.2022.849698)
Supplement: Supplementary file 4 [file Table_4.docx]

**Supplementary TABLE 4** **| Mycotoxin production of some representative isolates.**

| **Isolate** | **Species** | **Mycotoxin content (µg/g)** | | | | | |
| --- | --- | --- | --- | --- | --- | --- | --- |
|  |  | **NIV** | **ZEN** | **DON** | **FB_1_** | **FB_2_** | **FB_3_** |
| LPS-LZ-01 | *F. meridionale* | 0.1012 ± 0.0010 | ND | ND | ND | ND | ND |
| ZY-SY-01 | *F. meridionale* | 0.0249 ± 0.0014 | ND | ND | ND | ND | ND |
| QXN-XR-01 | *F. meridionale* | ND | 0.1644 ± 0.0031 | ND | ND | ND | ND |
| GY-GSH-01 | *F. meridionale* | ND | 0.0593 ± 0.0022 | ND | ND | ND | ND |
| QXN-ZF-01 | *F. cortaderiae* | ND | ND | 2.8995 ± 0.0380 | ND | ND | ND |
| BJ-HZ-02 | *F. boothii* | ND | ND | 1.6005 ± 0.0678 | ND | ND | ND |
| GY-XF-02 | *F. verticillioides* | ND | ND | ND | 270.7281 ± 18.4581 | 117.1875 ±12.8425 | 143.0563 ± 8.2837 |
| QN-LD-03 | *F. verticillioides* | ND | ND | ND | 2269.3430 ± 24.0038 | 1326.5330 ± 27.6487 | 1038.0720 ± 62.6519 |
| GY-HX-03 | *F. miscanthi* | <LOD | <LOD | <LOD | 0.3653 ± 0.0149 | 0.1654 ± 0.0059 | <LOD |
| GY-HX-212 | *F. miscanthi* | <LOD | <LOD | <LOD | 0.1225 ±0.0036 | 0.0854 ± 0.0023 | <LOD |
| QDN-RJ-01 | *F. concentricum* | <LOD | <LOD | <LOD | 0.1352 ± 0.0025 | 0.1204 ± 0.0076 | <LOD |

LOD: Limit of detection (0.01); ND: Not detected.
